# A novel aldose-aldose oxidoreductase for co-production of D-xylonate and xylitol from D-xylose with *Saccharomyces cerevisiae*

**DOI:** 10.1007/s00253-015-6878-5

**Published:** 2015-08-12

**Authors:** Marilyn G. Wiebe, Yvonne Nygård, Merja Oja, Martina Andberg, Laura Ruohonen, Anu Koivula, Merja Penttilä, Mervi Toivari

**Affiliations:** VTT, Technical Research Centre of Finland Ltd., P.O. Box 1000, FI-02044 VTT Espoo, Finland

**Keywords:** D-Xylonic acid, Xylitol, D-Xylose, Glucose-fructose oxidoreductase, GFOR

## Abstract

An open reading frame CC1225 from the *Caulobacter crescentus* CB15 genome sequence belongs to the Gfo/Idh/MocA protein family and has 47 % amino acid sequence identity with the glucose-fructose oxidoreductase from *Zymomonas mobilis* (*Zm* GFOR). We expressed the ORF CC1225 in the yeast *Saccharomyces cerevisiae* and used a yeast strain expressing the gene coding for *Zm* GFOR as a reference. Cell extracts of strains overexpressing CC1225 (renamed as *Cc aaor*) showed some *Zm* GFOR type of activity, producing D-gluconate and D-sorbitol when a mixture of D-glucose and D-fructose was used as substrate. However, the activity in *Cc aaor* expressing strain was >100-fold lower compared to strains expressing *Zm gfor*. Interestingly, *C. crescentus* AAOR was clearly more efficient than the *Zm* GFOR in converting in vitro a single sugar substrate D-xylose (10 mM) to xylitol without an added cofactor, whereas this type of activity was very low with *Zm* GFOR. Furthermore, when cultured in the presence of D-xylose, the *S. cerevisiae* strain expressing *Cc aaor* produced nearly equal concentrations of D-xylonate and xylitol (12.5 g D-xylonate l^−1^ and 11.5 g D-xylitol l^−1^ from 26 g D-xylose l^−1^), whereas the control strain and strain expressing *Zm gfor* produced only D-xylitol (5 g l^−1^). Deletion of the gene encoding the major aldose reductase, Gre3p, did not affect xylitol production in the strain expressing *Cc aaor*, but decreased xylitol production in the strain expressing *Zm gfor*. In addition, expression of *Cc aaor* together with the D-xylonolactone lactonase encoding the gene *xylC* from *C. crescentus* slightly increased the final concentration and initial volumetric production rate of both D-xylonate and D-xylitol. These results suggest that *C. crescentus* AAOR is a novel type of oxidoreductase able to convert the single aldose substrate D-xylose to both its oxidized and reduced product.

## Introduction

D-Xylose, the main component of hemicellulose, is the second most abundant sugar in nature after cellulose and starch-derived D-glucose. D-Glucose is widely used in various applications and processes, whereas the use of the pentose sugar D-xylose is not yet fully established. New products and production processes are needed for efficient utilization of hemicellulose and D-xylose.

Various studies have shown possibilities for biotechnical conversion of D-xylose to ethanol, butanol, lactic acid, succinic acid, xylonic acid, xylitol, hydrogen, modified sugars (transglycosylation) or compounds derived from fatty acid metabolism (Dumon et al. 2012; Peng et al. 2012). These products are formed via metabolism by natural or engineered microbes, e.g. of species like *Escherichia coli*, *Corynebacterium glutamicum*, *Zymomonas mobilis*, *Saccharomyces cerevisiae* and *Scheffersomyces stipitis*. The first conversion step of D-xylose is mostly performed with either xylose isomerase (EC 5.3.1.5) converting D-xylose to D-xylulose, D-xylose reductase (EC 1.1.1.307 or EC 1.1.1.21) converting D-xylose to xylitol, or D-xylose dehydrogenase (EC 1.1.1.175 or EC 1.1.1.179) converting D-xylose to D-xylonate. These products can be used as such or converted further chemically or enzymatically. Xylitol and D-xylonate, the reduced and oxidized products of D-xylose, are the simplest conversion products with established or demonstrated applications (cf. D-xylulose). Xylitol is a widely used sweetener with, e.g. anti-caries properties. Xylitol is currently mainly produced by chemical reduction of D-xylose with a nickel catalyst, but D-xylose can also be reduced to xylitol using an aldose reductase enzyme in vitro or in vivo, e.g. in yeast (Nidetzky et al. 1996; Winkelhausen and Kuzmanova 1998; Hallborn et al. 1991). D-Xylose dehydrogenases or oxidases oxidize D-xylose to D-xylonate, which has applications, e.g. as a chelator or as a hydrogel modifier (Millner et al. 1994; Chun et al. 2006; Zamora et al. 2000). D-Xylonate is produced by various bacteria or as described more recently by recombinant microbes, such as *E. coli*, *S. cerevisiae*, *Kluyveromyces lactis* or *Pichia kudriavzevii* (Nygård et al. 2011; Toivari et al. 2012a, b; Toivari et al. 2013; Liu et al. 2012; Cao et al. 2013). The reactions for enzymatic oxidation or reduction of D-xylose have so far been performed with separate enzymes using NADP^+^/NADPH or NAD^+^/NADH (or alternatively FAD^+^/FADH or PQQ) as cofactors. Whole cell conversions are usually preferred over in vitro enzymatic conversions for redox-linked reactions because regeneration of the cofactor in large scale in vitro systems is expensive. However, it is also well known that introduction of a redox reaction affects the cellular cofactor pool and that optimal production may necessitate further engineering of redox metabolism, use of a co-substrate or increased aeration. Some enzymes, such as the glucose-fructose oxidoreductase (EC 1.1.99.28), have a bound cofactor and can perform both oxidation and reduction reactions without interfering with the cellular redox balance (Piersma et al. 1997; Zachariou and Scopes 1986). Glucose-fructose oxidoreductase activity generates two products, an acid and an alcohol, which could be applicable, e.g. in polymer synthesis.

The glucose-fructose oxidoreductase of *Z. mobilis* has been characterized and its crystal structure solved. It has remained the only characterized enzyme of this unique oxidoreductase type, in spite of its interesting properties with bound cofactor, a redox neutral reaction cycle and two products.

Our long-term interest in pentose sugar utilization has led to searches for novel D-xylose-converting enzymes, isomerases, reductases, and dehydrogenases. Some D-xylose dehydrogenases belong to the Gfo/Idh/MocA enzyme family, which also includes the glucose-fructose oxidoreductase of *Z. mobilis*. During studies on D-xylose dehydrogenases (Toivari et al. 2012a, b), we encountered an open reading frame (ORF) CC1225 from the fresh water bacterium *C. crescentus* (synonym *Caulobacter vibrioides*) automatically annotated as a glucose-fructose oxidoreductase or as a D-xylose dehydrogenase. To evaluate whether this ORF (CC1225) is involved in D-xylose conversion, we expressed it in *S. cerevisiae*, using the well-characterized GFOR of *Z. mobilis* as a reference enzyme.

## Materials and methods

### Strain construction

*S. cerevisiae* strain CEN.PK 113-17A (H2802; MATα, *ura3-52 HIS3 leu2-3*/*112 TRP1 MAL2-8* ^*c*^ *SUC2*) (Entian and Kötter 1998) was used as the parental strain for all genetic modifications. The strains used in the study are listed in Table 1.

**Table 1:** *S. cerevisiae* strains used in this study. w/o ss; without signal sequence

Strain	Strain number	Genotype or parent strain + plasmid (B-number)
CEN.PK113-17A	H2802	MATα, *ura3-52 HIS3 leu2-3*/*112 TRP1 MAL2-8* ^*c*^ *SUC2*
control	VTT-C-15930	H2802 + B1181
*Cc aaor*	VTT-C-15928	H2802 + B3353
*Cc aaor xylC*	VTT-C-15929	VTT-C-15928 + B3574
*Zm gfor*	VTT-C-15933	H2802 + B3895
*Cc aaor* w/o ss	VTT-C-15935	H2802 + B4023
*Zm gfor* w/o ss	VTT-C-15934	H2802 + B4022
∆*GRE3*	VTT-C-15927	H2802 ∆*GRE3*
∆*GRE3* control	VTT-C-15931	H2802 ∆*GRE3* + B1181
*Cc aaor* ∆*GRE3*	VTT-C-15932	H2802 ∆*GRE3* + B3353

The putative glucose-fructose oxidoreductase encoding gene from *C. crescentus* CB15 (CC1225, AAK23207.1, NCBI), hereafter called *Cc aaor* (gene) or *Cc* AAOR when referring to the enzyme), was obtained as a synthetic gene, codon optimized for *S. cerevisiae*, deposited sequence KR269738 (Gene Art, Germany). Similarly, the gene encoding the glucose-fructose oxidoreductase of *Z. mobilis* (Q07982.2, NCBI), hereafter called *Zm gfor* (gene) or *Zm* GFOR (when referring to the enzyme), was obtained as a synthetic gene, codon optimized for *S. cerevisiae*, deposited sequence KR269739 (Genscript, New Jersey). The genes *Cc aaor* and *Zm gfor* were ligated into the *Bgl*II site, between the *PGK1* promoter and terminator, of YEplac195 + *PGK1*PT (B1181), generating plasmids B3353 and B3895, respectively. Both genes contained signal sequences for periplasmic translocation. The signal sequences were removed by amplifying the genes with primers CCTACTGAATTCAGATCTACAATGGCTCAACCAGGTAGAAAATTG and TTACCTGGATCCAGATCTTCACAATTTAACAGTTCTA for *Cc aaor* and ATCGTAGATCTAAGTTACTATGGCAACTTTACCTGCCGGTGC and TTGCAGAGATCTTCATTAGTAAC for *Zm gfor* using plasmids B3353 and B3895 as templates. The resulting *Cc aaor* and *Zm gfor* fragments were cloned into the B1181 expression vector as described above, resulting in plasmids B4023 and B4022, for *Cc aaor* and *Zm gfor*, respectively.

Plasmids B3353, B3895, B4023 and B4022 were introduced into *S. cerevisiae* strain H2802 to generate strains VTT-C-15928, VTT-C-15933, VTT-C-15935 and VTT-C-15934, respectively (Table 1). A control strain was created by introducing plasmid B1181 to *S. cerevisiae* CEN.PK 113-17A (H2802), resulting in strain VTT-C-15930. Plasmid B3353 was also introduced into the Gre3p-deficient strain VTT-C-15927 (H3613, Toivari et al. 2010), resulting in strain VTT-C-15932. A control strain was created by introducing plasmid B1181 to Gre3p-deficient strain VTT-C-15927, resulting in strain VTT-C-15931. Plasmid B3574 expressing xylonolactone lactonase encoding gene *xylC* from *C. crescentus* (Toivari et al. 2012a, b) was transformed to strain VTT-C-15928 resulting in strain VTT-C-15929.

### Media, culture conditions and measurement of biomass

Yeast strains were cultured in 50 ml of modified synthetic complete medium lacking uracil (SC-ura, modified from Sherman 1983) in 250-ml Erlenmeyer flasks, at 250 rpm, 30^○^C. D-Glucose was used as a carbon source and D-xylose was added in concentrations indicated in the text. Calcium carbonate (CaCO_3_, 1 % *w*/*v*) was used to buffer the medium in flask cultures. In bioreactors, yeasts were grown in 500 ml medium (SC-ura) in Multifors bioreactors (max. working volume 500 ml, Infors HT, Switzerland) at pH 5.5, 30 °C, 1 volume air [volume culture]^−1^ min^−1^ (vvm) and 500 rpm agitation with 2 marine impellors (Toivari et al. 2010). The pH was maintained constant by addition of 2 M NaOH or 1 M H_2_PO_4_. Clerol antifoaming agent (Cognis, France, 0.08–0.10 μl l^−1^) was added to prevent foam formation.

Biomass was measured as optical density (OD) at 600 nm (OD_600_) and/or as dry weight. For cultures containing CaCO_3_, OD was determined by diluting the sample 6-fold with 4 N HCl to solubilize CaCO_3_. Subsequent dilutions, if needed, were made in water. For dry weight, samples were collected in 2-ml pre-dried, pre-weighed microcentrifuge tubes, washed twice with equal volume distilled water and dried at 100 °C.

### Bioinformatic analysis

**Fig. 1:**
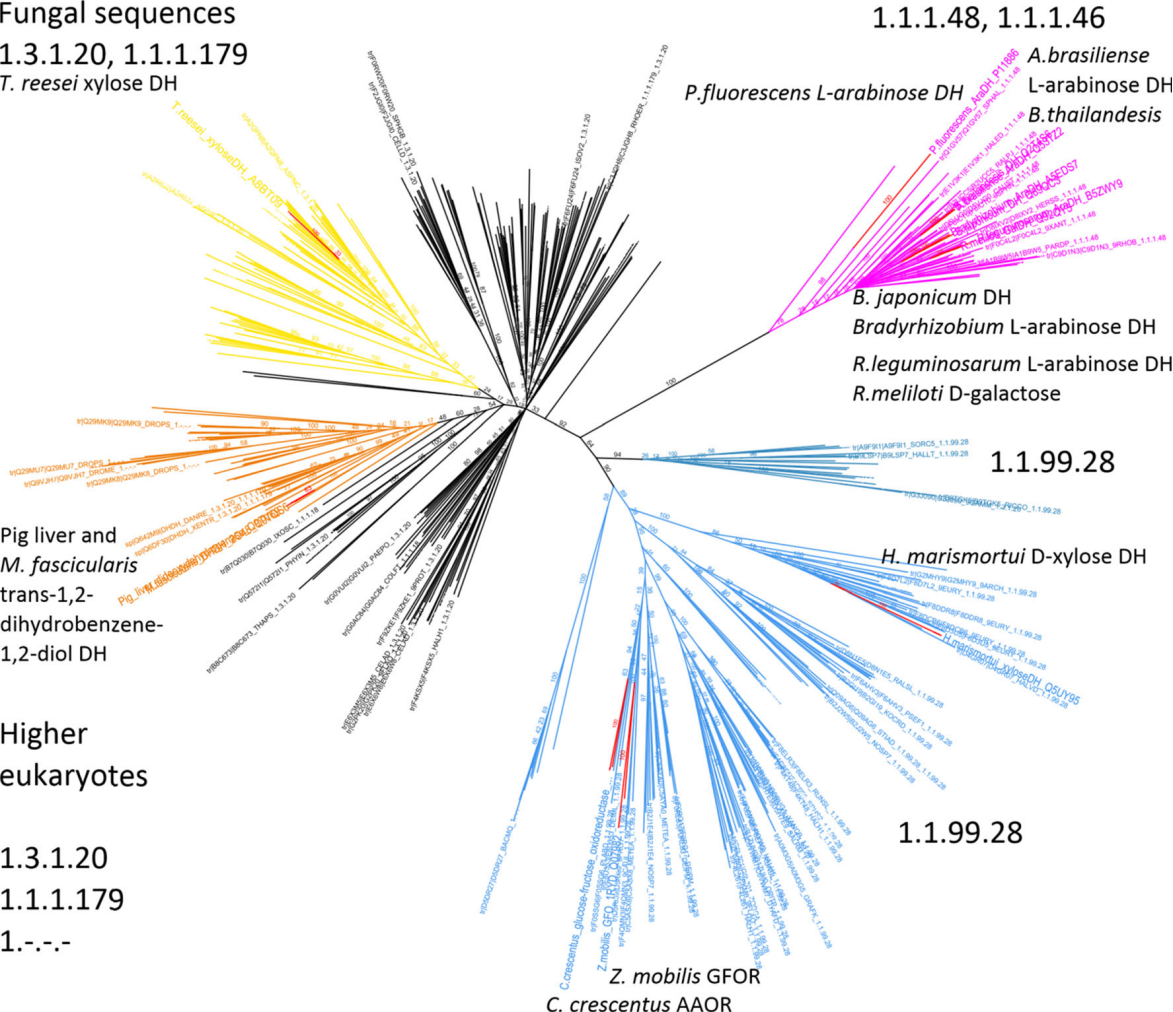
A phylogenetic tree of aldose dehydrogenase enzymes of the Gfo/Idh/MocA family. The *pink branch* contains L-arabinose and D-galactose dehydrogenases. The *light blue branch* contains bacterial and archaeal GFOR, *Cc* AAOR and archaeal D-xylose dehydrogenases. The *orange branch* contains trans-1,2-dihydrobencene-1,2-diol dehydrogenases and D-xylose dehydrogenases from higher eukaryotes. The *yellow branch* contains fungal sequences, including the *T. reesei* D-xylose dehydrogenase. Sequence names are only shown for sequences with verified or tentative E.C. number annotation in the UniProt database

The similarity relationship of the *Cc* AAOR protein coding sequence to the Gfo/Idh/MocA family, especially to D-xylose and L-arabinose dehydrogenases, was mapped from a protein BLAST search (http://blast.ncbi.nlm.nih.gov). *Cc* AAOR sequence together with *Zm* GFOR, and the known D-xylose or L-arabinose, or putative L-arabinose dehydrogenase sequences of the Gfo/Idh/MocA family were used as query sequences in a BLAST search against the UniProt database (http://www.uniprot.org/). The query protein sequences were as follows: *C. crescentus* AAOR (Q9A8X3), *T. reesei* D-xylose DH (A8BT09), *H. marismortui* xylose DH (Q5UY95), *Z. mobilis* GFOR (Q07982), *A. brasiliense* L-arabinose DH (Q53TZ2), *R. leguminosarum* L-arabinose DH (B5ZWY9), *Bradyrhizobium* L-arabinose DH (A5EDS7), *P. fluorescens* L-arabinose DH (P11886), *B. thailandesis* DH (Q2T4S6), *B. japonicum* DH (Q89QC3), *R. meliloti* galactose DH (Q92QY5), *M. fascicularis* trans-1,2-dihydrobenzene-1,2-diol DH (Q9TQS6) and pig liver trans-1,2-dihydrobenzene-1,2-diol dehydrogenase (Q9TV69) (the identifiers refer to UniProt, the Universe protein resource, available at http://www.uniprot.org/). The search, conducted in October 2013, returned 2454 unique sequences. To remove redundancy among the retrieved sequences, they were clustered using BLASTclust (ftp://ftp.ncbi.nih.gov/blast/documents/blastclust.html) to groups that are 80 % identical along 70 % of the sequence length. After clustering, 424 sequences remained. The query sequences were added and the set of 437 sequences were aligned using CLUSTALW (www.ebi.ac.uk/clustalw/). Non-full length sequences were removed, and the multiple sequence alignment of the remaining 426 sequences was used as an input to phylogenetic tree reconstruction using the Geneious Tree Builder with default parameters and with resampling (consensus of 100 trees). The tree is visualized in Fig. 1.

### Analytical procedures

The extracellular compounds D-xylonate, xylitol, ethanol, glycerol, acetate, D-glucose and D-xylose were analysed by HPLC on a fast acid analysis column linked to an Aminex HPX-87H column (BioRad Labs, USA) with 2.5 mM H_2_SO_4_ as eluent and a flow rate of 0.5 ml min^−1^. The column was maintained at 55 °C. Peaks were detected using a Waters 410 differential refractometer and a Waters 2487 dual wavelength UV (210 nm) detector. When D-xylonate was present, D-xylose concentrations were estimated by subtraction of the D-xylonate concentrations (detected by UV) from the combined D-xylose and D-xylonate peak detected by RI. Extracellular D-xylonate concentrations were also measured using the hydroxymate method (Lien 1959) as described by Toivari et al. (2010). Capillary electrophoresis as described in (Rovio et al. 2010; Turkia et al. 2013) was used for measuring D-gluconate concentrations in enzyme assays. Xylitol or D-sorbitol, from the enzymatic reduction reactions with D-xylose or D-fructose, respectively, were measured with a D-sorbitol/xylitol kit (Megazyme, Ireland) using a Konelab 20X automated analyser (Thermo Scientific, MA).

### Measurement of enzyme activity

Enzyme activities were measured from cell extracts. Cell extracts were prepared with acid-washed glass beads in 2-ml tubes with screw-on caps with a roughly 1:1:1 ratio of glass beads, cell pellet (wet) and lysis buffer (50 mM sodium phosphate buffer pH 6.8, 1X cOmplete EDTA-Free Protease Inhibitor, Roche). The protein concentration of cell extracts was determined using the Bio-Rad Protein Assay kit, based on Bradford 1976 (Bradford 1976), with IgG as a standard.

D-Xylose dehydrogenase activity, with either NAD^+^ or NADP^+^ as a cofactor and 100 mM D-xylose as substrate, was measured as described by Berghäll et al. (2007). For the measurement of glucose-fructose oxidoreductase activity, D-glucose (400 mM) and D-fructose (800 mM) were used as co-substrates (comparable to concentrations in Zachariou and Scopes 1986). Alternatively D-xylose (10 mM) alone was used as substrate. All reactions (400 μl total volume) were carried out at 22 °C in 100 mM potassium phosphate buffer pH 6.5. The reactions with 400 mM D-glucose and 800 mM D-fructose were sampled at 0, 15, 30 and 60 min; reactions with 10 mM D-xylose were sampled at 0, 1, 2, and 3 h. Reactions were stopped by heating at 100 °C for 10 min. Xylitol, D-sorbitol or D-gluconate was measured as conversion products as described above. Activity was calculated as micromoles per minute product produced (per g of protein in cell extract), based on the concentration of D-sorbitol, xylitol or D-gluconate produced (depending on the substrate(s)).

## Results

### Sequence similarities and genomic features of the *C. crescentus* aldose-aldose oxidoreductase

The predicted amino acid sequence of the open reading frame from *C. crescentus* CB15 with locus tag CC1225, hereafter called aldose-aldose oxidoreductase, *Cc* AAOR, has 47 % amino acid sequence identity with the glucose-fructose oxidoreductase from *Z. mobilis* (*Zm* GFOR). It is annotated as a putative glucose-fructose oxidoreductase or NAD(P)-dependent oxidoreductase belonging to the Gfo/Idh/MocA family (PFAM family domains: PF01408, N-terminal and PF02894, C-terminal), members of which typically have a NAD(P)-binding Rossmann fold. This family also contains L-arabinose/D-galactose dehydrogenases, D-xylose dehydrogenases and mammalian trans-1,2-dihydrobenzene-1,2-diol dehydrogenases with D-xylose dehydrogenase activity (Fig. 1). The D-xylose dehydrogenase xylB of *C. crescentus*, cloned and characterized earlier (Stephens et al. 2007; Toivari et al. 2012a, b), belongs to the family of short chain dehydrogenases (SDR) and shares little sequence similarity with enzymes of the Gfo/Idh/MocA family.

The ORF CC1225 corresponds to 366 predicted amino acids, including a putative periplasmic targeting signal sequence of 27 amino acids which was identified with the SignalP programme (www.cbs.dtu.dk/services/SignalP (Petersen et al. 2011)). The signal sequence region is shorter than that of the *Z. mobilis* GFOR, which consists of 52 amino acids. Sequences similar to the protein potentially coded by CC1225 of *C. crescentus* CB15 are also found in genomes of *C. crescentus* NA1000 (100 % identity) and *Caulobacter segnis* ATCC 21756 (85 % identity) (NCBI BLASTP 2.2.29 (Altschul et al. 1997)). Interestingly, the annotated sequence from *C. crescentus* NA1000 has an additional sequence of 32 amino acids at its N-terminus, rendering it more similar in length to the signal sequence of *Z. mobilis* than the sequence from *C. crescentus* CB15. A DNA sequence coding for a similar region of amino acids is also found upstream of the predicted start codon of the CC1225 ORF in the CB15 strain, and in *C. segnis* ATCC 21756, suggesting the possibility that the translation start codon is not correctly predicted in the ORFs of *C. segnis* and *C. crescentus* CB15. The ORF CC1225 appears to be part of a putative operon of three genes in the genome of *C. crescentus* CB15, including the predicted genes encoding gluconolactonase (CC1224), glucose-fructose oxidoreductase (CC1225) and a protein with unknown function (CC1226).

### Expression and enzymatic activity of the putative oxidoreductase AAOR of *C. crescentus* and GFOR of *Z. mobilis* in *S. cerevisiae*

The *aaor* (the CC1225 open reading frame) from the *C. crescentus* CB15 genome was codon optimized for *S. cerevisiae* and cloned under the *PGK1* promoter in a multicopy vector. Additionally, *Cc aaor* without the predicted 27 amino acids of the signal sequence was cloned. The *gfor* gene of *Z. mobilis* was cloned to the same vector backbone with and without its signal sequence. The plasmids were introduced into the *S. cerevisiae* lab strain CEN.PK 113-17A or alternatively into its derivative, deficient in the aldose reductase Gre3p (Table 1).

No D-xylose dehydrogenase activity was detected in the cell extracts of *S. cerevisiae* strains expressing either *Cc aaor* or *Zm gfor* using the described D-xylose dehydrogenase assay (Berghäll et al. 2007), which measures reduction of NAD(P)^+^. However, cell extracts of strains expressing either the *gfor* of *Z. mobilis* or the *aaor* of *C. crescentus* showed production of D-sorbitol and D-gluconic acid in the glucose-fructose oxidoreductase activity assay (Table 2). The activity of *Zm* GFOR in the cell extract from strain VTT-C-15933 was 120 or 40 fold higher than that of *Cc* AAOR in the cell extract from strain VTT-C-15928, when measured as D-sorbitol or D-gluconate, respectively (Table 2). Although *Cc* AAOR activity was clearly lower than *Zm* GFOR activity, it was still higher than the background activity of the control strain, which showed no sorbitol and a 11 fold lower amount of D-gluconate production compared to the *Cc aaor* expressing cell extract. To test the activity with substrate concentrations that are more likely to be found inside the cell, a concentration of 10 mM was selected for measuring the activity with D-xylose. Conversion of D-xylose as a single substrate at the chosen concentration was not detectable with cell extracts containing *Zm* GFOR, whereas cell extracts with *Cc* AAOR showed 0.12–0.20 μmol min^−1^ mg protein^−1^ activity, when measuring xylitol as the product (Table 2).

**Table 2:** Oxidoreductase activity in *S. cerevisiae* strains expressing *Cc aaor* or *Zm gfor*. Conversion of D-glucose + D-fructose or D-xylose alone in crude cell extracts was measured with different substrate concentrations with either D-sorbitol and D-gluconate as products (D-glucose + D-fructose as substrates) or xylitol as a product of D-xylose conversion, respectively. Units are μmol min^−1^ [mg protein]^−1^ in cell extract

Strain	H-number	Glu (400 mM) Fru (800 mM) D-Sorbitol	Glu (400 mM) Fru (800 mM) D-Gluconate	Xyl (10 mM) Xylitol
*Cc aaor*	VTT-C-15928	0.66 ± 0.01	1.40 ± 0.27	0.12 ± 0.00
*Cc aaor* ∆*GRE3*	VTT-C-15932	0.54 ± 0.19	0.86 ± 0.74	0.20 ± 0.01
*Zm gfor*	VTT-C-15933	72 ± 2.5	49 ± 7.7	0.01 ± 0.00
Control	VTT-C-15930	0.00 ± 0.00	0.13 ± 0.01	0.00 ± 0.00

### Co-production of D-xylonate and xylitol from D-xylose with *S. cerevisiae* strains expressing *Cc aaor*

**Fig. 2:**
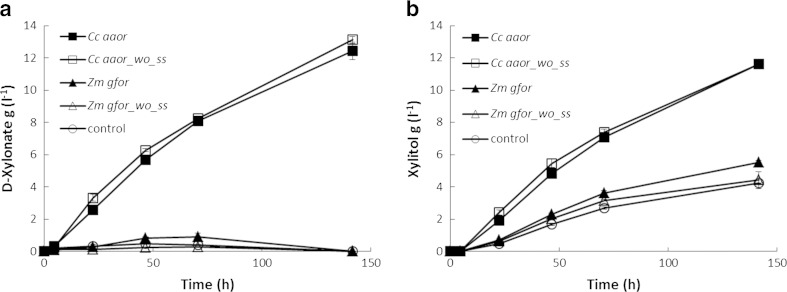
Production of D-xylonate and xylitol in flask cultures of *S. cerevisiae* strains expressing *aaor* of *C. crescentus* or *gfor* of *Z. mobilis*. Medium containing D-glucose and D-xylose was buffered with CaCO_3_

*S. cerevisiae* strains expressing *Cc aaor* or *Zm gfor* were grown in flasks in SC-ura medium containing 10 g D-glucose l^−1^, 20 g D-xylose l^−1^ and 10 g CaCO_3_ l^−1^. The strain expressing *Cc aaor* produced 12.5 g D-xylonate l^−1^ and 11.5 g xylitol l^−1^, whereas the strain expressing *Zm gfor* and the control strain both produced 5 g xylitol l^−1^ but no D-xylonate (< 1 g l^−1^) (Fig. 2).

To assess whether the signal sequence present in the originally cloned *Cc aaor* and *Zm gfor* genes affected D-xylonate or xylitol production, yeast strains expressing *Cc aaor* and *Zm gfor* versions without the signal sequences were also grown in flasks buffered with CaCO_3_. Again, the strain expressing *Cc aaor* (VTT-C-15935) produced 12 g D-xylonate l^−1^ and 11 g xylitol l^−1^ from 26 g D-xylose l^−1^ in 140 h, whereas the strain expressing *Z. mobilis gfor* produced only xylitol, 5 g l^−1^ (Fig. 2).

### Co-production of xylitol and D-xylonate with *S. cerevisiae* strains expressing *aaor* of *C. crescentus* in bioreactor cultures

**Fig. 3:**
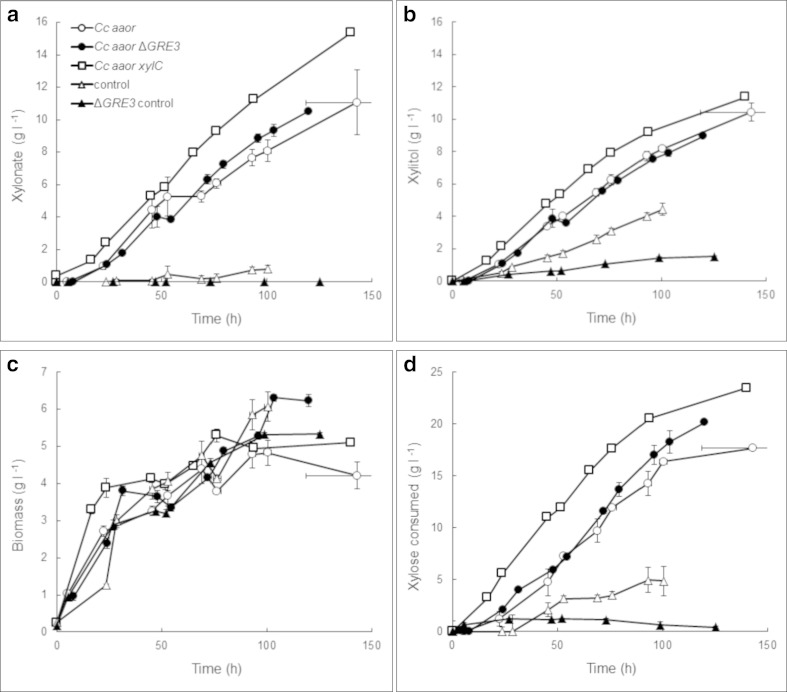
Production of **a** D-xylonate, **b** xylitol, **c** biomass and **d** consumption of D-xylose by *S. cerevisiae* control (*triangles*) and strains expressing *Cc aaor* (*circle* and *squares*) in wild type (*open symbols*) or Δgre3 (*solid symbols*) strains. Cells were grown in bioreactors with D-xylose and D-glucose, pH 5.5, 30 °C, 500 rpm agitation, 1 vvm aeration. Error bars show SEM (*n* = 2–6)

When strains expressing *Cc aaor* (with or without the native aldose reductase encoding gene *GRE3*) were grown in bioreactor culture, 10.8 ± 0.8 g D-xylonate l^−1^ and 9.7 ± 0.5 g xylitol l^−1^ were produced from 20 to 25 g D-xylose l^−1^ (Fig. 3). The molar ratio of D-xylonate and xylitol in the culture supernatant was 1.0 ± 0.0 throughout the cultivation. Strains expressing *Cc aaor* produced twice as much xylitol as the control strain (with *GRE3*) throughout the first 90 h cultivation, and still had 80 % more xylitol than the control after 100 h. Deletion of *GRE3* did not affect xylitol production when *Cc aaor* was expressed, but reduced xylitol production in its absence (Fig. 3). The *Cc aaor* strains produced D-xylonate and xylitol at volumetric rates of 108 mg D-xylonate l^−1^ h^−1^and 88 mg xylitol l^−1^ h^−1^. The D-xylose uptake rate was 192 mg D-xylose l^−1^ h^−1^, which was 2.8 times faster than the parent strain without *Cc aaor* but with an intact *GRE3* gene. Neither expression of *Cc aaor* nor deletion of *GRE3* affected biomass production in *S. cerevisiae* under these conditions (Fig. 3).

Both D-xylonate (53 ± 6 mg g^−1^) and xylitol (54 ± 12 mg g^−1^) accumulated intracellularly in *S. cerevisiae* expressing *Cc aaor*. The intracellular D-xylonate concentration was higher in the ∆*GRE3 Cc aaor* strain than in the strain with a functional Gre3p (87 ± 6 mg g^−1^) but the intracellular xylitol concentration was similar (50 ± 4 mg g^−1^, *p* > 0.05). The control strain accumulated 41 ± 4 mg g^−1^ xylitol, similar (*p* > 0.05) to that observed in the *Cc aaor* expressing strains, whereas deletion of *GRE3* reduced this significantly (*p* < 0.05) to 25 ± 1 mg g^−1^.

Co-expression of the D-xylonolactone lactonase encoding gene *xylC* from *C. crescentus* with the *Cc aaor* gene resulted in slightly increased production of both D-xylonate (15 g l^−1^) and xylitol (11 g l^−1^, Fig. 3), at initial volumetric rates of 140 and 130 mg l^−1^ h^−1^, respectively. Intracellular concentrations of D-xylonate (63 ± 7 mg g^−1^) and xylitol (43 ± 4 mg g^−1^) were similar to those observed in the strains lacking the lactonase.

## Discussion

**Fig. 4:**
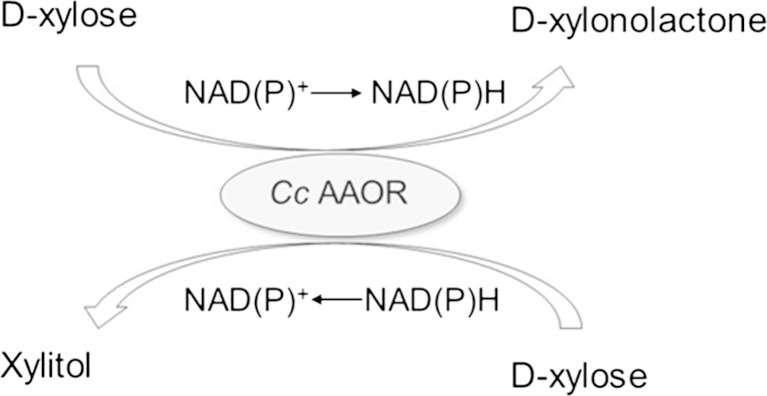
Suggested oxidation-reduction reactions on D-xylose, carried out by the *Cc* AAOR enzyme from *C. crescentus*

The newly identified enzyme (CC1225, named *Cc* AAOR) from *C. crescentus* was shown to convert in vitro the single aldose sugar D-xylose to D-xylitol, without an added cofactor. When the *Cc aaor* gene was expressed in *S. cerevisiae*, D-xylonate and xylitol were produced from D-xylose in equimolar concentrations. These results suggest that *Cc* AAOR functions as an aldose-aldose oxidoreductase (Fig. 4), being able to both reduce and oxidize D-xylose as a single substrate. The GFOR from *Z. mobilis* that belongs to the same protein family, does not need an added cofactor, but requires two substrates, preferably an aldose (D-glucose) and a ketose (D-fructose) sugar. The difference between these two oxidoreductases was clear when they were expressed in *S. cerevisiae* and cultured in the presence of D-xylose: the *Zm* GFOR containing strain did not produce D-xylonate from D-xylose and only similar concentrations of xylitol as the parental strain. To the best of our knowledge, *Cc* AAOR is the first single substrate aldose-aldose reductase to be described.

*Z. mobilis* GFOR has been intensively studied and it is so far the only confirmed glucose-fructose oxidoreductase. It does not have very close homologues based on sequence similarity: the closest homologues are 53 % identical, while *Zm* GFOR and *Cc* AAOR share 47 % sequence similarity. *Cc* AAOR has higher sequence similarity to other putative enzymes (max. 65 %), which may also be interesting aldose-aldose oxidoreductases.

Both *Cc* AAOR and *Zm* GFOR possess a signal sequence of the TAT-pathway (twin-arginine translocation pathway). It is known that *Zm* GFOR is folded as a pre-protein containing the signal sequence and is active both with and without it (Loos et al. 1993). The signal sequence for *Cc* AAOR (derived from *C. crescentus* CB15) is shorter than that of *Zm* GFOR, and *Cc* AAOR was shown to be similarly active both with and without the signal sequence, when expressed in *S. cerevisiae*. Based on comparison of genome sequences from related bacterial species, it is possible that the native *Cc* AAOR from *C. crescentus* CB15 strain or from other *Caulobacter* species have longer signal sequences. With *Zm* GFOR the signal sequence is cleaved during the translocation to the periplasmic space. We anticipate that the TAT-pathway is not functional in yeast and that the enzymes are located in the cytosol when expressed in *S. cerevisiae*. The physiological function of AAOR in *C. crescentus* remains currently unclear.

The *Zm* GFOR enzyme has high *K*_m_ values, and high substrate concentrations (400 mM to 1 M) have been used to measure its activity (Zachariou and Scopes 1986; Zhang et al. 2009). The *Z. mobilis* GFOR is located in the periplasmic space where the high concentrations needed may occur. In this study, the *Zm gfor* and *Cc aaor* genes were expressed intracellularly and thus, lower substrate concentrations were also tested with the single substrate D-xylose. When high concentrations of D-glucose and D-fructose were used as substrates, both *Zm* GFOR and *Cc* AAOR containing cell extracts showed activity in vitro, i.e. production of D-gluconic acid and D-sorbitol via the oxidation-reduction reaction cycle, respectively. However, the activity of *Cc* AAOR was more than 100-fold lower compared to the activity of *Zm* GFOR. Interestingly, when a single substrate, D-xylose, was used in assessing the enzyme activities, *Cc* AAOR had 10-fold higher activity compared to *Zm* GFOR. During the 3-h incubation an average of 180 and 17 mg xylitol ^−1^ was formed by *Cc* AAOR and *Zm* GFOR, respectively. It is possible that the *Cc* AAOR enzyme is inhibited by high substrate concentrations and/or that it reacts only with D-glucose (analogous to D-xylose) in the glucose-fructose oxidoreductase assay. D-Xylonate was not detected in the CE measurements performed, possibly because the system does not detected lactone forms and no lactonase enzyme was present. Measurements with optimized reaction conditions, different substrates and substrate concentrations, and more sensitive analytical methods are needed to further define the activity of this enzyme.

Despite the low *Cc* AAOR activity on D-xylose in vitro, a reasonable production rate of D-xylonate and xylitol from D-xylose were obtained when *Cc aaor* was expressed in *S. cerevisiae*. D-Xylonate and xylitol were formed in equimolar concentrations, further supporting a mechanism of a single substrate aldose-aldose oxidoreduction for *Cc* AAOR (Fig. 4). There was no evidence of D-gluconate being produced from the D-glucose provided for growth, indicating that glycolysis efficiently competed with *Cc* AAOR for this hexose sugar. *S. cerevisiae* has several aldose reductases which result in production of xylitol from D-xylose. When *Cc aaor* was expressed in a *S. cerevisiae* strain deficient in the major aldose reductase Gre3p, again equal concentrations of D-xylonate and xylitol were formed, whereas without *Cc aaor* expression xylitol production was reduced by ~70 %, further supporting the suggested mechanism of action of *Cc* AAOR. It seems that *Cc* AAOR can efficiently compete with Gre3p for the in vivo conversion of D-xylose.

The volumetric production rates and concentrations of D-xylonate obtained with *S. cerevisiae* expressing *Cc aaor* were much higher than those of D-xylonate-producing yeast strains expressing NADP^+^-dependent D-xylose dehydrogenases of the Gfo/Idh/MocA family (xyd1 of *T. reesei* or SUS2DD from pig liver, Toivari et al. 2012a, b). Compared to the rate of D-xylonate production obtained with the strain expressing the NAD-dependent *C. crescentus* D-xylose dehydrogenase *xylB* of the short-chain dehydrogenases family in *GRE3*-deficient strain, the D-xylonate production rate of the *Cc aaor* strain was about half (0.11 compared to 0.23 g l^−1^ h^−1^), reflecting the co-production of xylitol with the D-xylonate. The xylose consumption rates of *Cc aaor* or *xylB* expressing strains were comparable (0.19 and 0.21 g l^−1^ h^−1^, respectively). With *Cc aaor* expressing strains the xylitol production rate and final titre were much higher compared to any of the D-xylose dehydrogenase expressing strains. As all D-xylose provided was converted to the two products D-xylonate and xylitol, the total yield of products on D-xylose was 1.

The future bioeconomy needs novel solutions for production of chemicals in a sustainable way. New enzymes and engineered microbial strains enable development of versatile biorefinery concepts. The biosphere contains an enormous reserve of enzymes, of which we currently only apply a few. The present paper describes a novel aldose-aldose oxidoreductase, which is assumed to contain a bound cofactor and thus provides a self-sustained oxidation-reduction catalyst, able to produce both an oxidized and reduced product from a single substrate. Such enzymes provide interesting options for both in vitro and in vivo applications. The *Cc* AAOR enzyme was successfully used for conversion of the abundant, but today still inefficiently exploited biomass sugar D-xylose to two products, D-xylonate and xylitol, in a single reaction by a single yeast strain. With increasing xylitol demand and potential interest in various applications of D-xylonate this enzyme provides an interesting option for their co-production. The differences between the *Cc* AAOR and the *Zm* GFOR are yet to be determined in detail, but the differences in action are likely due to structural differences, particularly in the N-terminal region. We are currently in the process of characterizing the properties and determining the crystal structure of *C. crescentus* AAOR to obtain a more thorough understanding of the enzyme and to be able to fully exploit its potential.
